# Cardiac valve disease: an unreported feature in Ehlers Danlos syndrome arthrocalasia type?

**DOI:** 10.1186/1824-7288-38-65

**Published:** 2012-11-16

**Authors:** Daniela Melis, Gerarda Cappuccio, Virginia Maria Ginocchio, Giorgia Minopoli, Maurizia Valli, Massimiliano Corradi, Generoso Andria

**Affiliations:** 1 Department of Paediatrics, Federico II University, Via Sergio Pansini 5, Naples, 80131, Italy; 2 Department of Biochemistry, University of Pavia, Pavia, Italy; 3 Department of Mother and Child, University of Verona, Verona, Italy

**Keywords:** Ehlers Danlos syndrome type VII B, Macrocephaly, Cardiac valve regurgitation

## Abstract

Ehlers Danlos syndrome (EDS) athrocalasia type (type VII), is characterized by joint hypermobility, skin hyperextensibility and tissue fragility. No heart involvement has been reported. Two forms have been described: type VII A and VII B. The abnormally processed collagen α2(I) and the skipping of the exon 6 in COL1A2 gene are typically detected in EDS type VII B. We describe a seven-year old female, with a phenotype consistent with EDS type VII B and a diagnosis further confirmed by biochemical and molecular analyses. Cardiac ultrasound showed normal data in the first year of life. When she was 5 years old, the patient developed mitral valve regurgitation, and aortic and tricuspidal insufficiency at 7 years of age. To our knowledge, this is the first report of cardiac valvular involvement in EDS VII B. This feature probably has been underreported for the limited follow-up of the patients. Echocardiography might be warranted in the clinical assessment of EDS VII patients.

## Introduction

Ehlers Danlos syndrome (EDS) is a group of inherited connective tissue disorders. Six major forms can be distinguished on the basis of specific clinical features [1]. Over the last years, the molecular basis of several new EDS variants have been identified; these studies demonstrated the involvement of not only collagens but also other ECM-related molecules in determining EDS phenotype, refining and expanding the Villefranche classification [2].

EDS arthrocalasia type (formerly types VIIA and B), an autosomal dominant form, is very rare: about 40 cases have been reported [3]. EDS type VII is due to a defective processing of type I collagen synthesis. EDS type VII A is due to the disruption of procollagen chain α1(I), encoded by COL1A. EDS type VII B is due to the abnormality of α2(I), procollagen chain encoded by *COL1A2*.

The molecular defect associated with EDS type VII B is remarkably homogeneous and result in exon 6 skipping or genomic deletion of exon 6 *COL1A2* gene.

The main clinical features of EDS type VII B patients include: severe generalized hypermobility, recurrent joints subluxations, bilateral hip dislocation. Hyperextensibility of the skin, easy bruising, tissue fragility, hypotonia, kyphoscoliosis have also been reported.

Structural cardiovascular alterations have been described in cases of classical and hypermobile EDS [4]. Mitral valve prolapse is a manifestation of patients affected by vascular EDS [5]. Mitral regurgitation, and mitral valve prolapse, are reported in EDS kyphoscoliotic type [6]. Cardiac valvular involvement has never been reported in EDS arthrocalasia type.

We describe a seven-year and five-month old female patient affected by EDS type VIIB and showing mitral, aortic and tricuspidal valve regurgitation.

## Case report

The patient, a female, was the first child of healthy, non consanguineous parents, with a negative family history for genetic diseases. Foetal activity during gestation was normal.

She was born at 40 weeks of gestation by caesarean section, due to lack of labour progression. At birth, her weight was 3350 g (50^th^ centile), her length was 50 cm (50^th^ centile) and her head circumference was 38 cm (>95^th^ centile). Apgar score was 7-8, at 1 and 5 minutes of life, respectively. At birth, the infant showed marked axial hypotonia, atrial septal defect, ostium secundum type and bilateral congenital hip dysplasia, confirmed by hip ultrasound.

**Figure 1:**
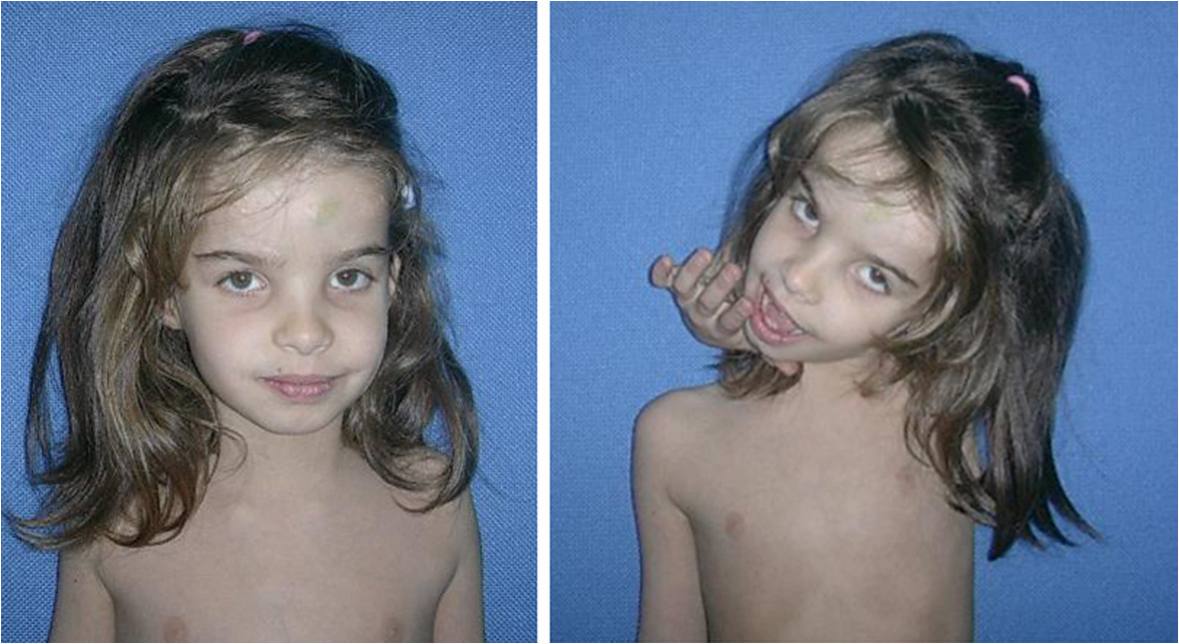
The child at 10 years of age.

**Figure 2:**
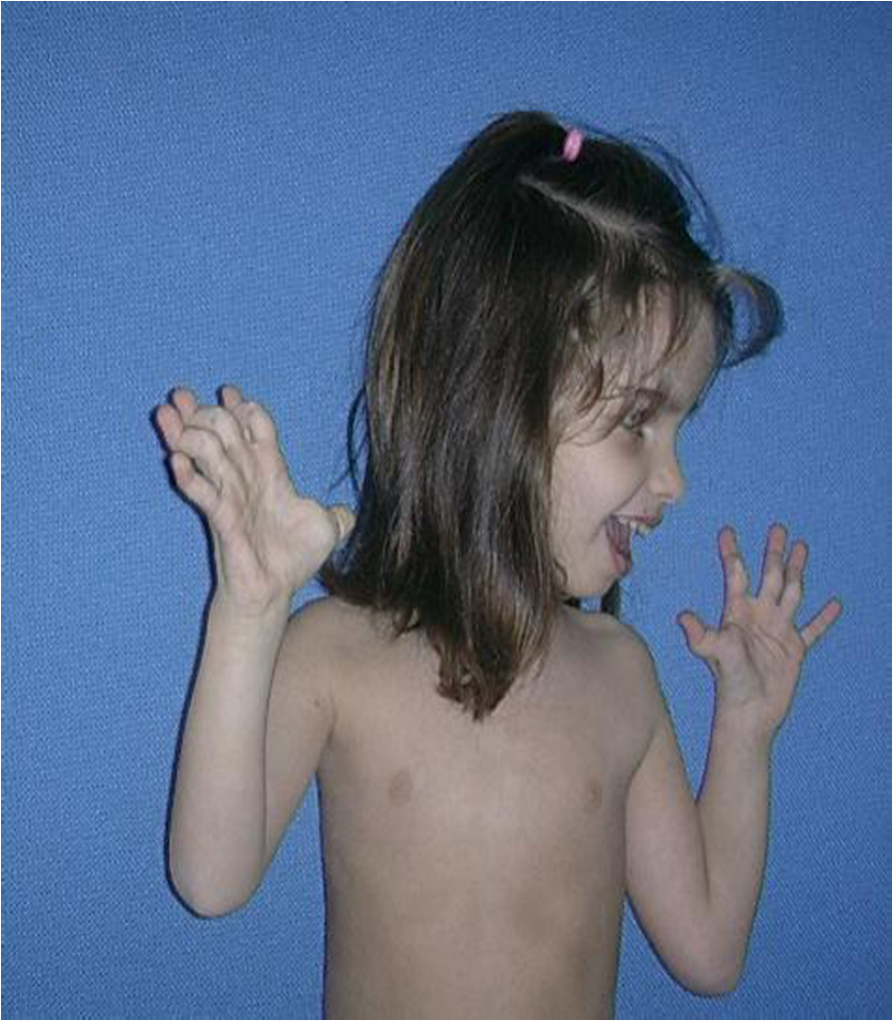
Marked joint laxity at wrist and fingers is notable.

**Figure 3:**
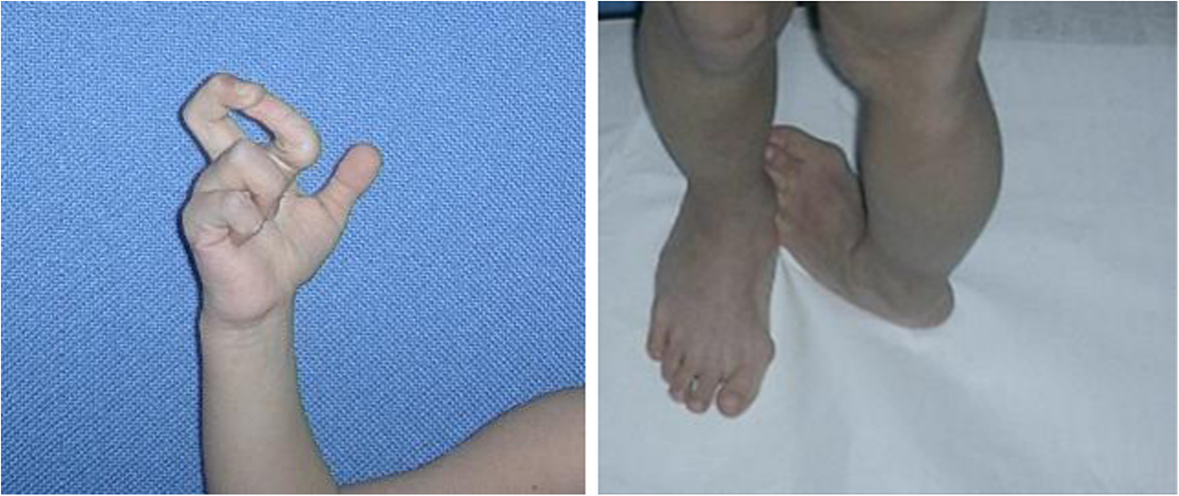
Joint laxity at ankle is evident.

At one month of age the patient was seen for the first time in the Clinical Genetics Unit of the Department of Paediatrics Federico II, University of Naples; physical examination, at that age, revealed macrocephaly (40.6 cm >95th centile), which was not a familiar feature, some dysmorphic facial features (prominent frontal bossing, posteriorly rotated and low-set ears, thick lips, high arched palate). Other clinical features included: large anterior and posterior fontanels, bilateral patellar subluxation, flat abducto-valgus deformities of the feet and bilateral hallux valgus, bilateral hip dislocation, redundant skin, especially on feet and hands, easy bruising and axial hypotonia (Figures 1, 2, 3). The diagnosis of EDS was suspected, therefore she underwent skin biopsy for biochemical study.

Collagen analysis with electrophoresis of dermal collagen showed an additional band migrating between the α1 (I), and α2 (I) chains of type I collagen with mobility of pro-α 1(I) collagen chains (pNα2(I)). The chains of other collagens (type III and V from the patient’s cells) were not affected. These findings were consistent with EDS type VII B disease.

Due to biochemical study results, the genetic test for *COL1A2* was performed.

Mutation analysis of fragments encompassing intron 5 to 6 of the *COL1A2*, showed a T-to-C transition at the second position T + 2 which altered the obligatory GT nucleotide to GC (g.8984 T > C, IVS6 + 2 T > C, Skip exon 6).

This mutation causes the skipping of the exon 6-encoded sequences and consequently the lack of the N-proteinase cleavage site from mutant proα chain of type I collagen. These tests definitively confirmed the clinical diagnosis.

Period follow-ups revealed a normal intellectual development; a mildly delay was observed in achiving motor milestones: she sitted up at 10 months, walked alone at 18 moths. Atrial septal defect closed spontaneously, proved by a further echocardiography, performed when she was three years old. An opthalmologic evaluation was performed at 4 years, showing normal data.

When she was 5 ^4/12^ years old, clinical examination revealed: joint hypermobility (score of 7/9) [1], bilateral patellar dislocations, velvety, redundant and easily stretching skin and bruising. She presented two keloids scars, one evident in the right mandibular region, another one in the frontal region. Auscultatory examination revealed a midsystolic murmure. She underwent cardiac ultrasound that reveal spontaneous closure of atrial septum defect, but thickening of mitral valve leaflets associated with mild mitral regurgitation. Left ventricular end-diastolic diameter resulted slightly increased (37 mm, normal values of 29-35 mm). Transthoracic echocardiograms using semiquantitative grading of valve regurgitation, were performed once a year. After two-year follow-up, combined valvulopathy, resulting in mitral, tricuspidal and aortic regurgitation, were detected. Mitral regurgitation was graded as moderate (Jet area/Left atrium area % correspond to 25%), while aortic and tricuspidal regurgitation were defined mild. Left ventricular ejection fraction was within normal reference (65%). Left ventricular mass (indexed to h^2.7^) was 0.4, aortic root diameter (assessed using body surface area (BSA)-adjusted) was 1.9 cm, both in normal ranges.

## Discussion

EDS VII B is a highly homogeneous syndrome. Diagnostic steps remain collagen screening and molecular analyses: pNα2(I) and the skipping of *COL1A2* exon 6 respectively, yield as markers of EDS type VII B. In the current patient pNα2(I), and a heterozygous mutation of the COL1A2 gene IVS6 + 2 T > C (g.8984 T > C) leading to the skipping of exon 6, were detected.

About 20 cases of EDS VII B have been previously described in the literature, three of them share the mutation IVS6 + 2 T > C.

The major clinical criteria for EDS VII B, consist in recurrent subluxation of the joints, bilateral hip dislocation and severe generalised joint hypermobility; minor features are skin hyperextensibility, tissue fragility, easy bruising, neonatal hypotonia, kyphoscoliosis, mild osteopenia [1].

The herein described patient shares with EDS VII phenotype most of the features (generalized hypermobility, recurrent subluxation of the joints, bilateral hip dislocation, hyperextensibility of the skin and easy bruising). Moreover the patient’s echocardiografic findings suggest a progressive cardiac valve disease. It is noteworthy that cardiac valvular disease has never been described in EDS type VII.

Aortic regurgitation, mitral valve prolapse and mitral regurgitation occur with increased frequency in patients with Marfan syndrome, Loeys-Dietz syndrome and other connective tissue disorders, including EDS. Cardiovascular complications (mitral valve prolapse and aortic root dilatation) have been previously reported in individuals with EDS vascular type, hypermobile EDS, EDS type VI [4-6].

The complete absence of proα2(I) chains, caused by homozygosity or compound heterozygosity for COL1A2 null mutations, define a subset of EDS patients, with hypermobility and a potential risk for cardiac valvular disease (aortic and mitral valve regurgitation are described), beyond the classical stigmata of EDS (skin and joint involvement) [7,8].

Rare patients have been reported with combined aortic, mitral and tricuspidal valve regurgitation and prolapse, and the definition of “multiple floppy valves syndrome” has been proposed [8]. These patients, beyond cardiac findings, show hyperextensive joints without any other stigmata of Marfan syndrome or EDS, suggesting an etiology due to an undefined collagen disorder [9].

These rare reports suggest that the integrity of α2(I) collagen chains, involved in EDS VII B, is strictly necessary for correct cardiac valvular development.

Type I collagen is the major fibrillar collagen, a prominent protein in connective tissue. Several collagen types as type I, III and V are expressed in heart valves leaflets and myocardium, crosslinking themselves and with proteoglycans and glycosaminoglycans [10,11].

Quantitatively minor collagens including type V collagen (causally involved in classical type EDS) and type III (implicated as a cause of vascular EDS) play an important role in type I collagen fibrillogenesis in the cardiovascular system [10,11].

“Multiple collagens cross-talking” is required for crucial architecture and biomechanical function in heart valves, whose dysregulation is associated with such changes in structure and with valvular thickening from the aberrant collagens deposition, defective development and failure of cardiovascular system function. A specific disorganization of collagen fibril assembly is detected in EDS type VII [7].

We speculate that the disruption of collagen dynamics in cardiac valve extracellular matrix homoeostasis may be a common mechanism resulting in valve dysfunctions.

The case reported discloses the potential cardiac involvement in patients affected by EDS type VII B. No further similar cases with EDS VII B have been described; it is noteworthy that the patients, already published with EDS type VII, did not receive a cardiological follow-up.

In conclusion we describe a patient with EDS VII B due to mutation IVS6 + 2 T > C, whose pathognomonic manifestation (congenital hip dislocation, joint laxity, subluxations, abnormal scars, easy bruising) have rapidly brought to the correct diagnosis, confirming that EDS type VII can be primarily a clinical diagnosis. Since we demonstrated a progressive valvular involvement a careful cardiac follow-up is warranted for EDS type VII patients, because of the risk for cardiac valvular problems in early childhood.

## Consent

Written informed consent was obtained from the parents of the patient for publication of this report and any accompanying images.

## Competing interests

The authors declare that they have no competing interests.

## Authors’ contributions

DM and GC, wrote the manuscript. GM and VMG were involved in the follow-up of the patient. MV and MC carried out the molecular genetic studies. All authors read and approved the final manuscript. GA critically revised the manuscript.
